# Supplementary figures and images for: A Systematic Evaluation of Interneuron Morphology Representations for Cell Type Discrimination
Source: Neuroinformatics. 2020 May 4;18(4):591–609. doi: 10.1007/s12021-020-09461-z (PMC7498503; doi:10.1007/s12021-020-09461-z)

**A**  
*Morphometric statistics*

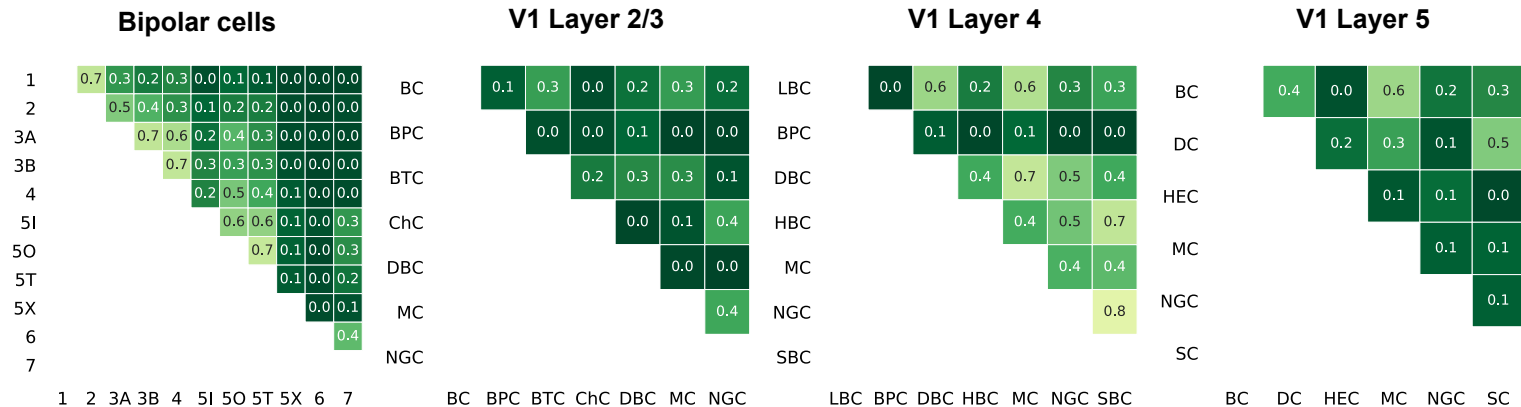

**B**  
*2D Persistence (z-proj)*

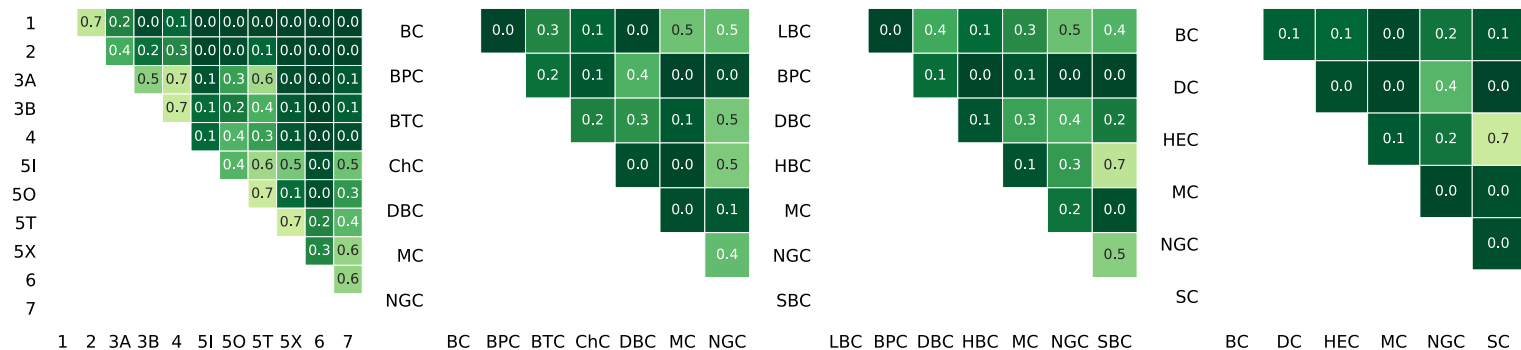

Supplement: Supplementary file 1 — (PDF 183 KB) [file 12021_2020_9461_MOESM1_ESM.pdf]

**A**

$$r^2 = 0.965$$
$$y = -0.48x^2 - 0.27x + 1.01$$

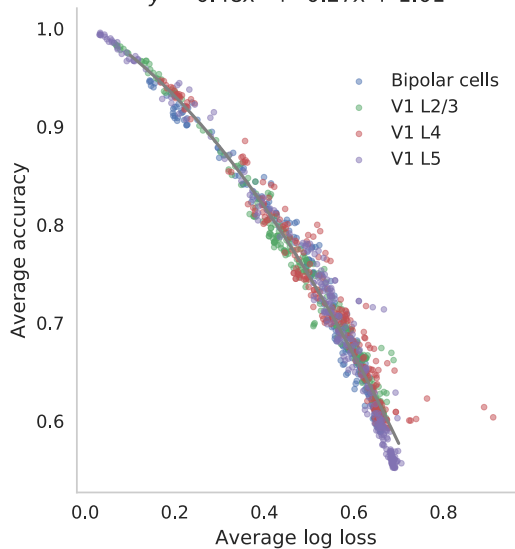**B**

$$r^2 = 0.950$$
$$y = -0.83x^2 - 0.32x + 1.00$$

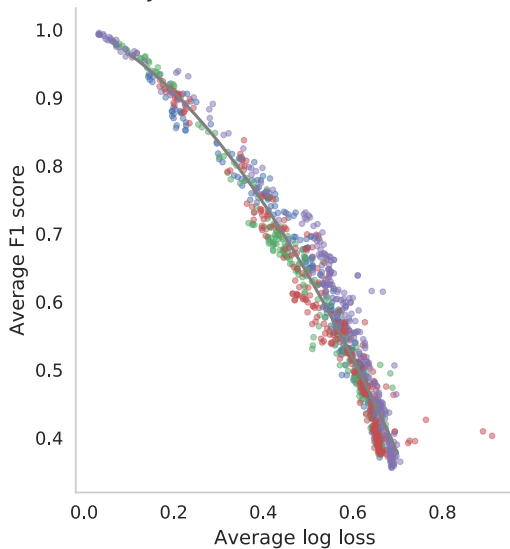**C**

$$r^2 = 0.952$$
$$y = -1.14x^2 - 0.67x + 1.02$$

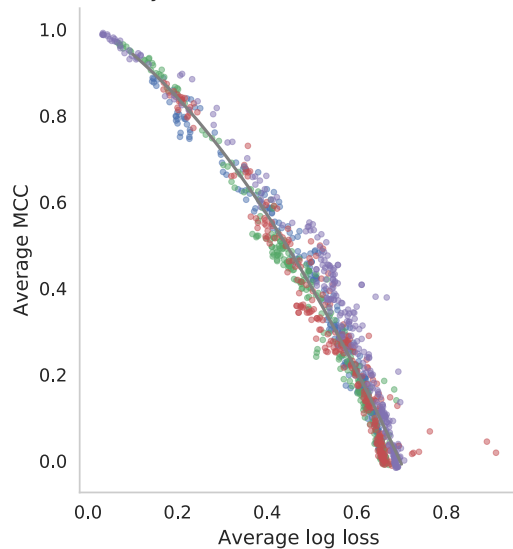

Supplement: Supplementary file 2 — (PDF 829 KB) [file 12021_2020_9461_MOESM2_ESM.pdf]

**A**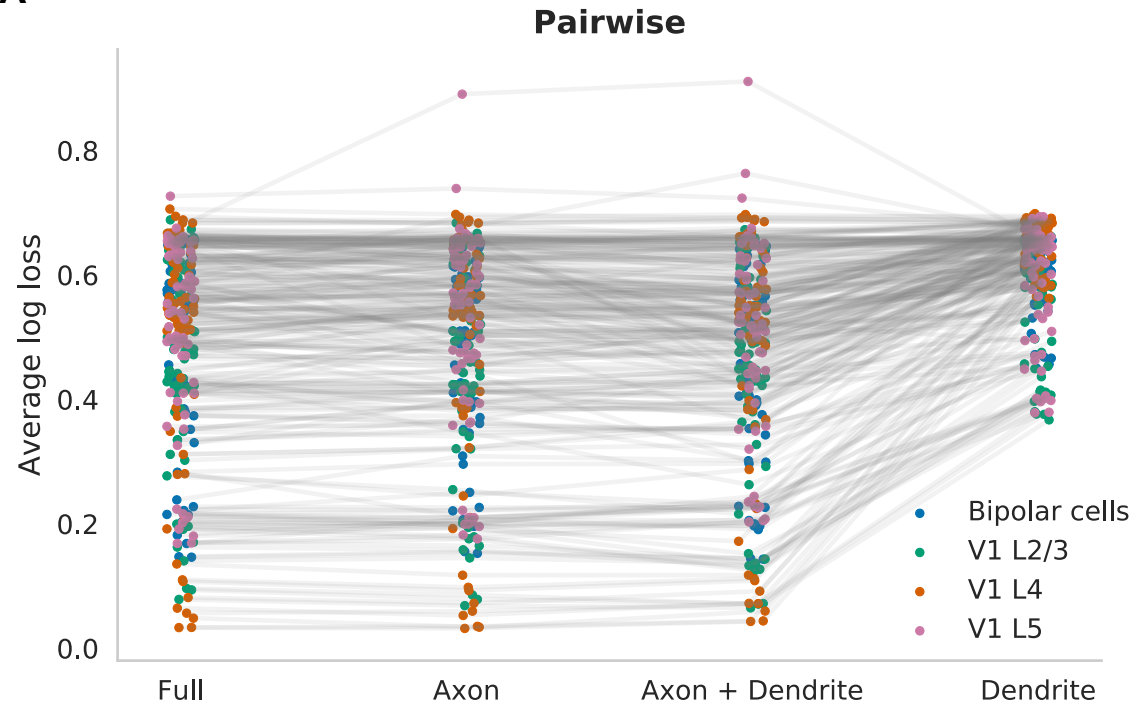**B**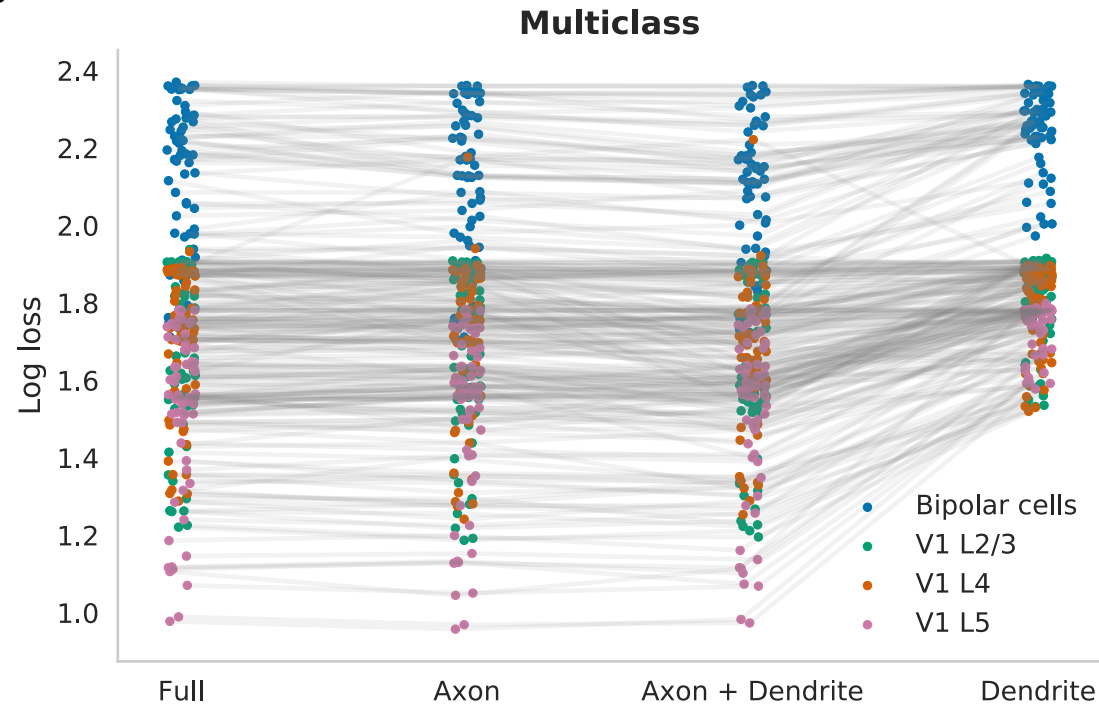

Supplement: Supplementary file 3 — (PDF 317 KB) [file 12021_2020_9461_MOESM3_ESM.pdf]

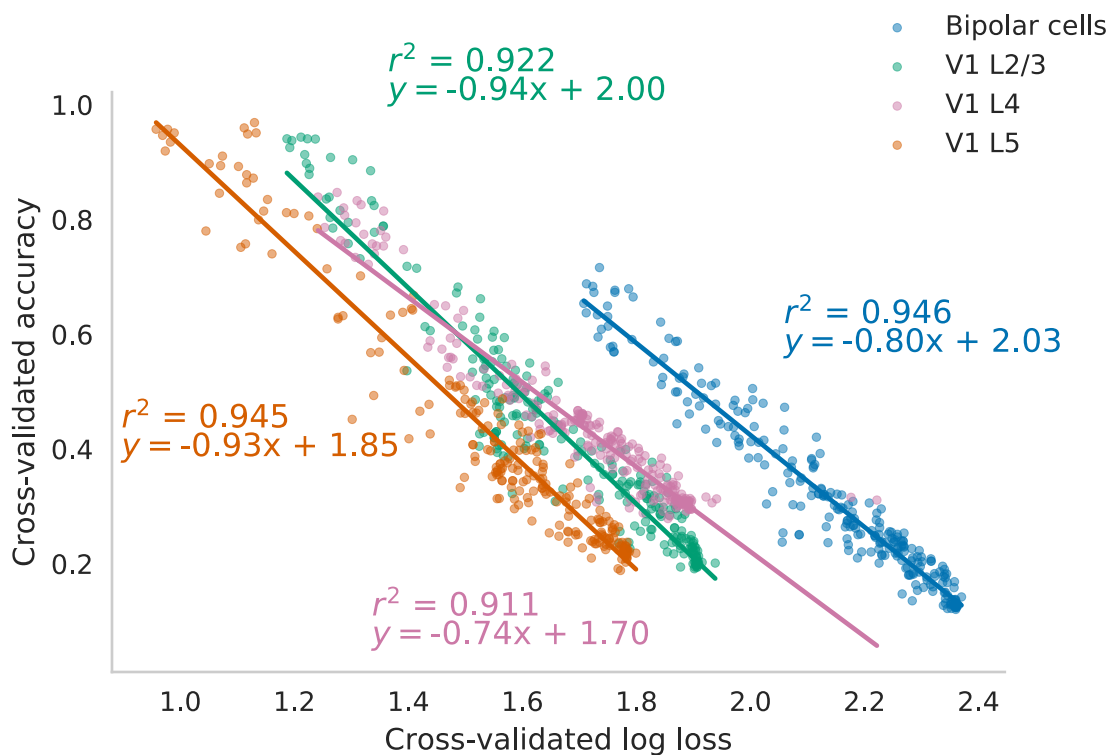

Supplement: Supplementary file 4 — (PDF 179 KB) [file 12021_2020_9461_MOESM4_ESM.pdf]

**Bipolar cells**

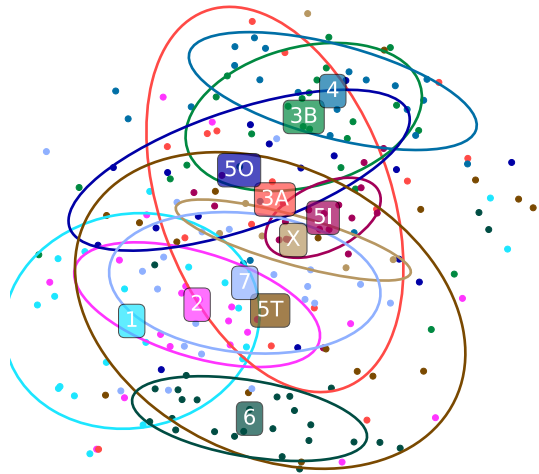

**V1 Layer 2/3**

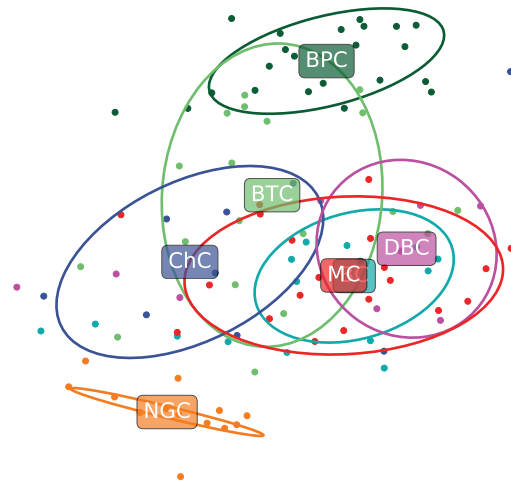

**V1 Layer 4**

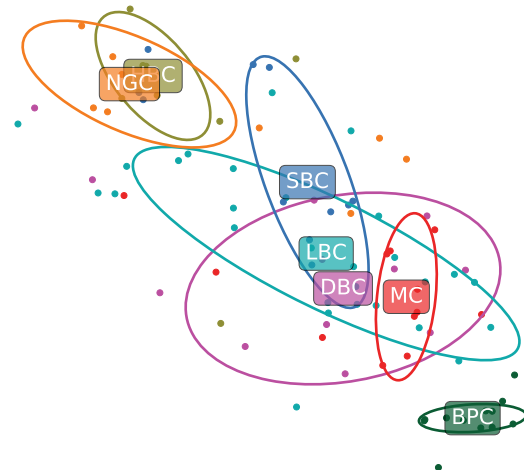

**V1 Layer 5**

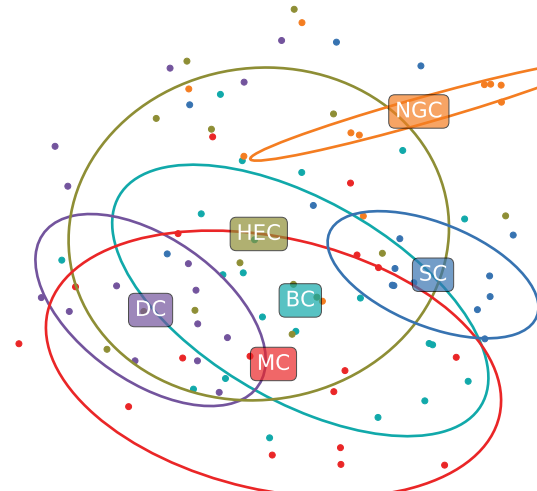

Supplement: Supplementary file 5 — (PDF 165 KB) [file 12021_2020_9461_MOESM5_ESM.pdf]

Bipolar cells

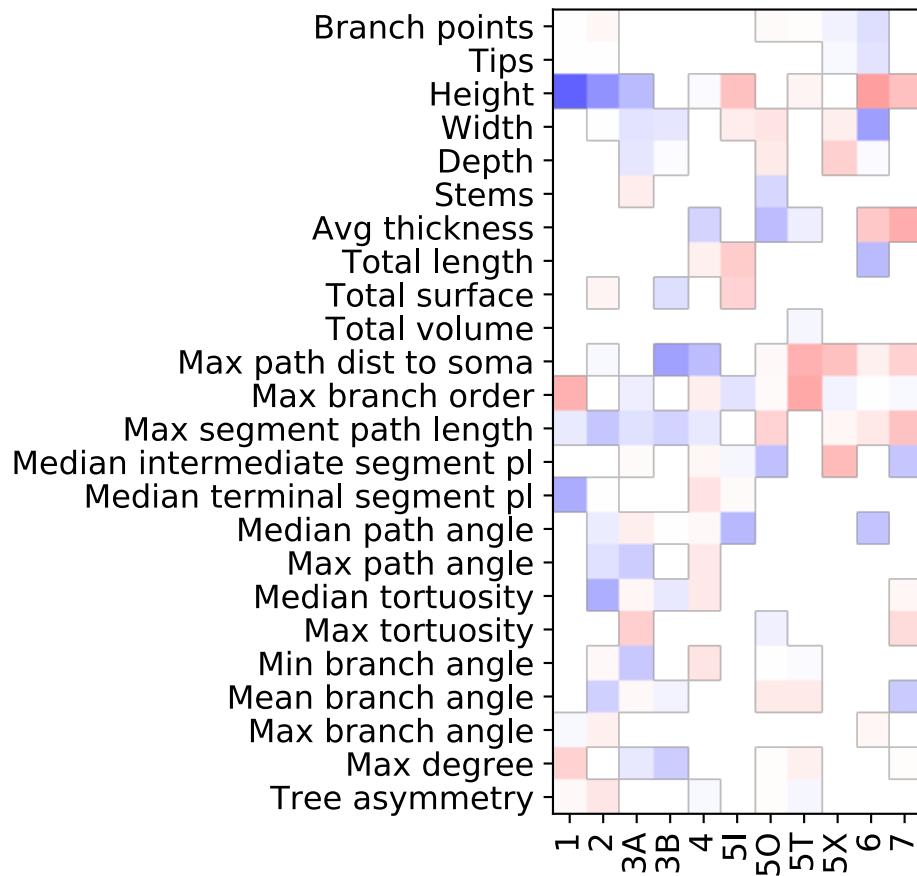

V1 Layer 2/3

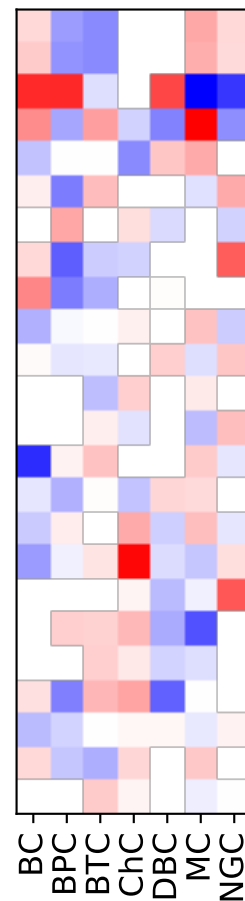

V1 Layer 4

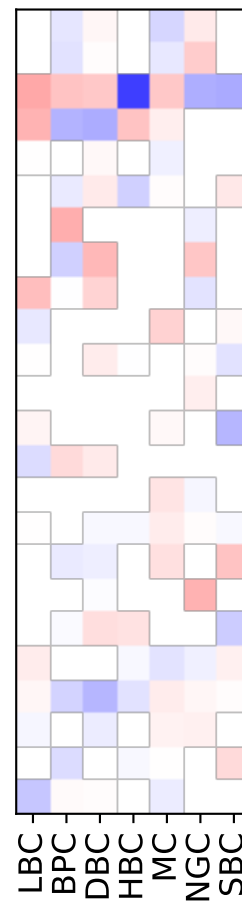

V1 Layer 5

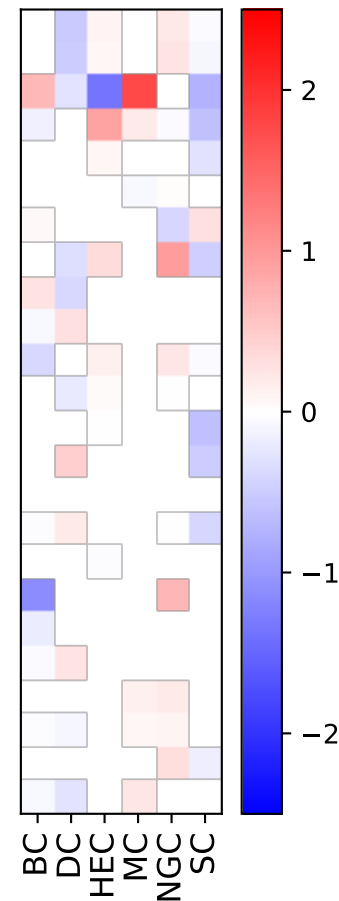

Supplement: Supplementary file 6 — (PDF 107 KB) [file 12021_2020_9461_MOESM6_ESM.pdf]
